# Transmit–Receive Module Diagnostic of Active Phased Array Antenna Using Side-Lobe Blanking Channel

**DOI:** 10.3390/s25216527

**Published:** 2025-10-23

**Authors:** Hongwoo Park, Wonjin Lee, Hyun Seok Oh, Seunghee Seo, Shin Young Cho, Hongjoon Kim

**Affiliations:** 1 Agency for Defense Development, Yuseong-gu, Daejeon 34141, Republic of Korea; hwpark43@add.re.kr (H.P.); wj_lee@add.re.kr (W.L.); ohs1231@add.re.kr (H.S.O.); seunghee@add.re.kr (S.S.); martin@add.re.kr (S.Y.C.); 2 Department of Electrical Engineering, Kyungpook National University, Sangyeok-dong, Daegu 41566, Republic of Korea

**Keywords:** airborne radar, active phased array antenna (APAA), fault detection/isolation, transmit–receive module (TRM), monopulse comparator, side-lobe blanking (SLB), peripheral probe

## Abstract

This article presents a diagnostic method for transmit–receive modules (TRMs) in an airborne active phased array antenna (APAA). Given the spatial constraints of airborne radar systems, the diagnostic functionality was implemented using the peripheral probe method. To minimize the space, cost, and time required for modifications to the existing APAA, the side-lobe blanking (SLB) channel was employed as the probe. To prevent TRM saturation and to determine the fault detection threshold, an APAA-level test was performed using a movable anechoic chamber. The coupling level between the SLB antenna and TRM was maintained between −70 dB and −20 dB. With the result of the APAA-level test, a budget analysis on the signal path was performed, and the input attenuation level was determined. The received signal power was estimated at −40 dBm to −20 dBm. Based on the estimation, the detection threshold was determined as −50 dBm. For the operation of the diagnostic function, simple detection logic and associated control timing is implemented in the radar processor. The effectiveness of the proposed diagnostic method was validated by several test activities, including an anechoic chamber, a rooflab facility, and an actual fighter. The test result shows good agreement with the expectations.

## 1. Introduction

The active phased array radar (APAR) is one of the most important sensors in modern military platforms such as aircraft, tanks, ships, and satellites. APAR systems provide diverse mission capabilities, including high-precision multi-target tracking, high-resolution ground mapping, and mode interleaving with various operational combinations [1,2,3,4,5,6]. Compared with mechanically scanned array radars, APARs significantly enhance platform mission capabilities due to their electronic beam steering agility [4,6].

These advantages arise from the agile beam scanning capability enabled by the transmit–receive modules (TRMs) embedded in the active phased array antenna (APAA). Each TRM is responsible for high-power transmission, low-noise amplification, and phase and amplitude modulation [7,8,9]. Typically, an APAR comprises hundreds or even thousands of TRMs.

During field operations, some TRMs may degrade or fail over time. Degradation refers to gradual changes in signal level or phase that do not necessarily require replacement, whereas failure denotes a complete loss of functionality caused by issues such as internal oscillation due to impedance instability or amplifier breakdown from excessive input power. In cases of failure, the APAA must be returned to a repair facility for TRM replacement [10,11].

Since an APAR continues to operate even when some TRMs are nonfunctional, system performance deteriorates gradually rather than abruptly, a characteristic known as graceful degradation [1,2,3,4,5,6]. To optimize maintenance scheduling and sustain radar performance, it is therefore essential to implement a built-in test (BIT) function capable of accurately identifying and isolating faulty TRMs [11,12]. By determining the number and locations of failed modules, the radar system can alert operators to maintenance needs, enabling informed decisions based on operational context. Moreover, an effective BIT function reduces the system’s overall life-cycle cost [13].

For TRM fault diagnostics, the peripheral antenna method is commonly used in ground-based and shipborne phased array radars [14,15,16,17,18,19]. This method injects a test signal into the TRMs through an external antenna, and the radar processor identifies faulty modules based on predefined threshold levels. While this approach effectively simplifies the signal injection network, it is challenging to integrate into airborne radar systems because it requires an external horn antenna and a dedicated signal generation module.

To overcome these limitations, this study proposes employing the side-lobe blanking (SLB) channel as a peripheral probe. The SLB channel, typically composed of a wide-beam antenna and transceiver circuitry, is used to suppress external clutter and jamming signals [20,21,22,23,24,25]. Previous studies on TRM BIT techniques have shown that antennas with wide beamwidths enable stable coupling between the probe and TRMs [26], making the SLB antenna well suited for this purpose. Furthermore, because the SLB transceiver provides an interface between the radar processor and the RF front end, the SLB channel inherently possesses the characteristics required for a peripheral probe.

By utilizing the existing SLB channel, the proposed approach eliminates the need for additional diagnostic antennas or signal generation circuits, thereby significantly reducing the cost and development time associated with implementing BIT functionality in existing APAR systems.

The effectiveness of the proposed method was verified using an APAR system deployed in an operational environment. The remainder of this article presents the fundamental design considerations and the step-by-step verification procedures used to evaluate the proposed diagnostic technique.

## 2. Design and Implementation

### 2.1. APAA System Architecture

**Figure 1:**
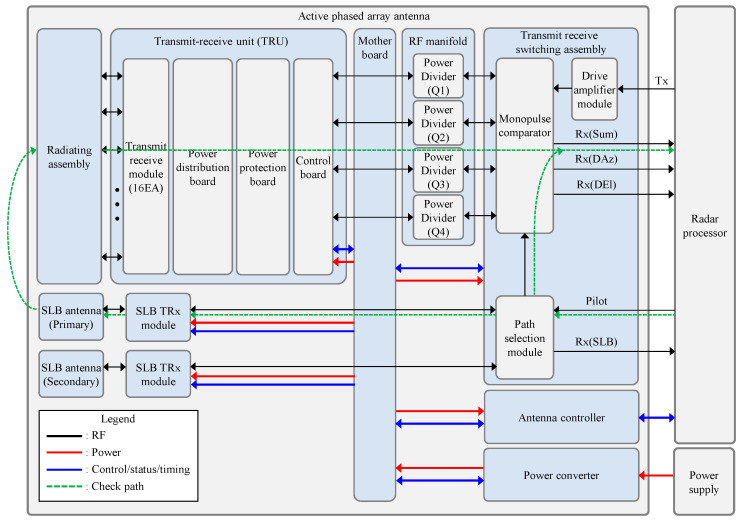
A block diagram of the APAA.

Prior to describing the proposed method, it is essential to understand the internal structure of the APAA. Figure 1 describes the block diagram of the APAA, and it required several submodules for its operation. The function of each submodule is summarized as follows.

- Radiating assembly was composed of large number of radiating elements for transmitting RF signals from TRMs and receiving RF signals reflected from the target.
- Transmit receive unit (TRU) was most important submodule inside the APAA. The TRU performed high-power amplification of the transmit signal and low-noise amplification of the received signal. For the electronical beam steering and the side-lobe-level control, the TRU had multifunction chips, which had an internal variable attenuator and a phase shifter. Above-mentioned functions were realized by sixteen TRMs inside TRU. Also, to support TRM functionality, the common module was composed of the power distribution board, the power protection board, and the control board, which were connected to TRMs.
- RF manifold distributed the high-power RF signal from the drive amplifier module and combined RF signals from TRUs. The RF manifold was composed of four power dividers for making four quadrant inputs to the monopulse comparator inside transmit receive switching assembly (TRSA).
- TRSA was mainly in charge of supporting high-power transmission by boosting signal from radar processor and forming monopulse-receiving channels (Sum, DAz, DEl) for high-precision target tracking. For an internal path check, the pilot port was used for injecting signal into the APAA. The path selection module inside TRSA selected signal injection path, and options are described as green dashed line in Figure 1. The signal path was determined by the command packet from the radar processor.

- SLB antenna was composed of open-ended waveguide horn antenna, which provided wide-beamwidth patten. For redundancy, the APAA had two SLB antennas.
- SLB TRx module provides the amplification of RF signal from TRSA and the low-noise amplification of received signal from the SLB antenna. For redundancy, the APAA has two SLB TRx modules.
- Motherboard distributed command data packet from antenna controller to TRUs and transmitted status data packet of TRU to antenna controller. Also, it distributed DC power from the power converter to other submodules.
- Antenna controller received command data from the radar processor and sent submodule status to the radar processor. Also, it sent a control packet and the timing signal controlling TRUs.
- Power converter converted the high input DC voltage to appropriate DC voltages for TRUs.

### 2.2. APAA Control Structure

**Figure 2:**
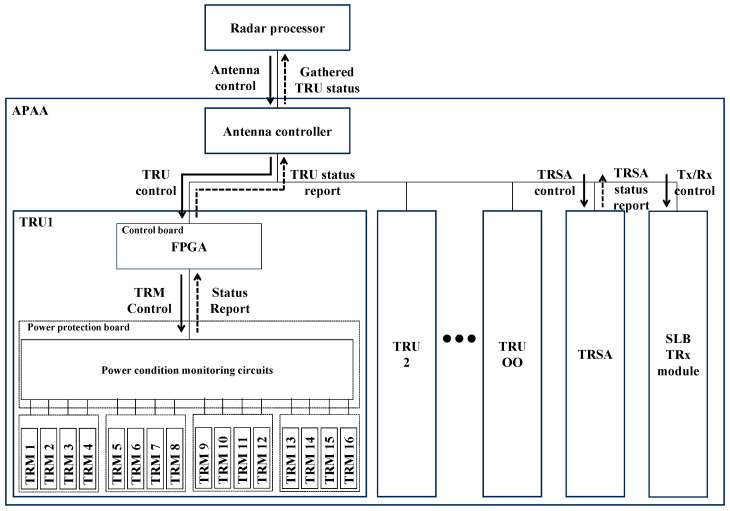
The command structure of the APAA.

Figure 2 depicts a simplified block diagram for explaining the control structure inside the APAA. For clarity, other irrelevant submodules such as power converters, motherboards, and RF manifold were omitted. TRU and TRSA had the internal field-programmable gate array (FPGA) to control inner components. The SLB TRx module was composed of simple elements such as regulators and RF amplifiers; thus, there was no need for inserting FPGA. Thus, the SLB TRx module was controlled directly by the antenna controller.

When the radar processor commanded APAA to check submodule status, the antenna controller received the command packet and sent a control message to submodules. When checking the receive path of TRMs, the TRU control board sequentially controlled each TRM. At the same time, the TRSA configured check path by controlling the path selection module. Also, the SLB TRx turned on the internal RF amplifiers to inject signal to the TRMs. Then, the radar processor sent an RF signal to the pilot port of the APAA and received signal from the Rx Sum port of the APAA. When checking the internal path of the TRSA, TRMs were not operating, and the path selection module configured did not switch to radiating signal through the SLB TRx module.

### 2.3. RF Budget Analysis

**Figure 3:**
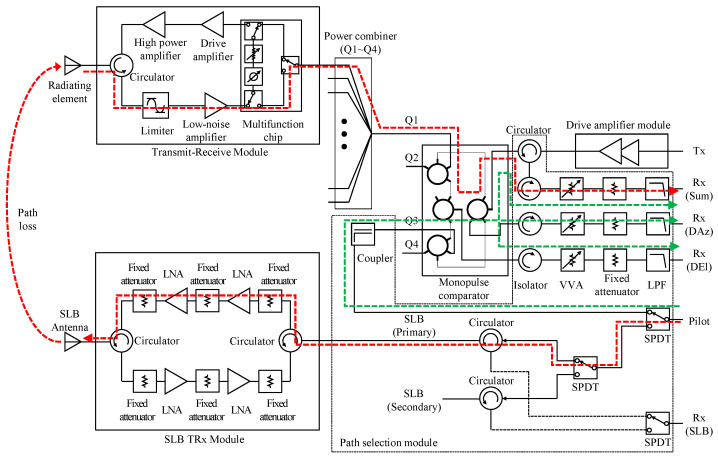
RF components inside the APAA (red dashed line: receive chain diagnostic path, green dashed line: TRSA internal check path).

Prior to designing fault detection algorithm, RF path analysis of the existing APAA was conducted for setting threshold for the failure determination. Figure 3 shows internal RF circuit block diagram inside the APAA, and the red dashed line indicates signal path for the receive path diagnostics. Besides the diagnostic path, the TRSA also had internal path for checking its internal passive circuit components, and it is described as green dashed line in Figure 3. The path selection module inside TRSA had three single pole dual through (SPDT) switches, and all of them were controlled by the command packet from the radar processor. Two SPDTs were used for selecting path for antenna diagnostics, and one SPDT was used for selecting SLB channel. Isolators and voltage variable attenuators (VVAs) provided isolation between Tx port and Rx port of the antenna, and VVAs were adjusted to provide maximum attenuation when the drive amplifier module was transmitting. The low pass filters (LPFs) prevented harmonic component input to the radar processor, and fixed attenuators were applied to provide impedance matching between circuit elements. The SLB TRx module had both transmit and receive path composed of identical circuit components.

Based on the block diagram shown in Figure 3, an RF budget analysis of the signal injection chain was conducted, and the results are summarized in Table 1. The “path loss” term in the table refers to the free-space loss between the SLB antenna and the radiating elements. The tabulated path loss represents a median value, while the measured variation according to TRM location is discussed in Section 2.4. The input signal level to the pilot port was limited to –33 dBm to prevent TRM saturation, given that the 1 dB gain compression point of a TRM was measured at –26 dBm. When the distance between the SLB antenna and a given TRM increased, the input signal level was adjusted proportionally to compensate for higher propagation loss. The maximum input level to the pilot port was set to 5 dBm.

### 2.4. Coupling Level Test and BIT Threshold Determination

**Figure 4:**
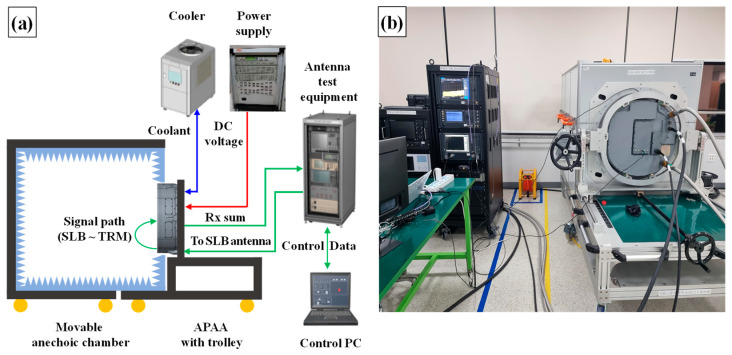
Coupling level test between SLB antenna and TRM: (**a**) test setup; (**b**) actual test scenario. (yellow circle: wheel, blue triangle: RF absorber).

To establish the BIT detection threshold, a coupling level test between the SLB antenna and the TRMs was conducted. The test configuration is illustrated in Figure 4. All measurements were performed inside a movable anechoic chamber to eliminate external interference. The APAA and its trolley were securely mounted to the chamber to minimize signal leakage from the antenna to the test facility. During the test, the antenna test equipment sent control packets to the antenna controller to activate one TRM at a time. Once a TRM was activated, a network analyzer within the antenna test equipment transmitted an RF signal to measure the S-parameters of the signal path between the input port of the SLB antenna and the Rx Sum port of the APAA. To prevent potential TRM damage caused by amplified signals from the SLB TRx module, both the TRSA and the SLB TRx module were excluded from the measurement path. A control PC configured and monitored the test equipment, while all measurement data were recorded on the same system. Adequate cooling and DC power were supplied to the APAA by a dedicated cooler and power supply unit.

**Figure 5:**
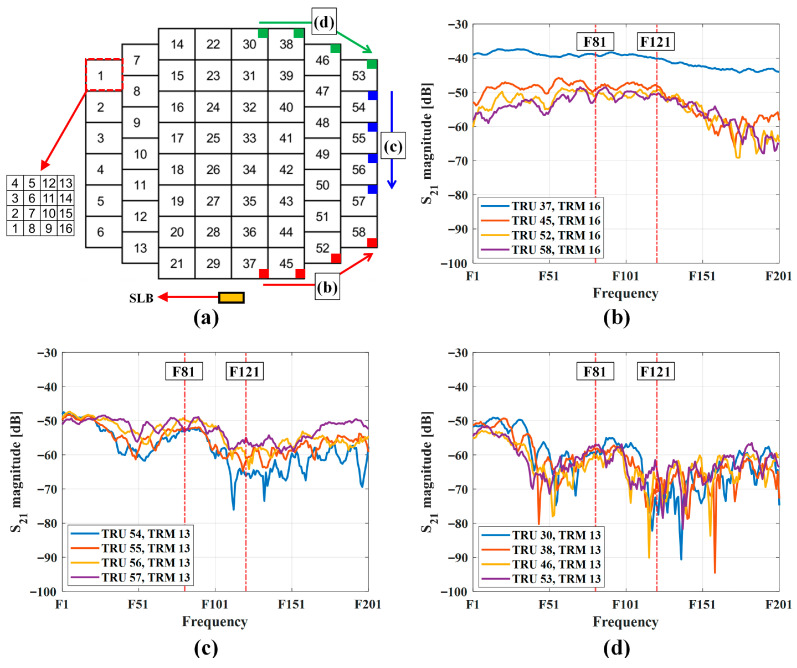
Coupling level test results for critical points. Red vertical lines (F81, F121) indicate selected BIT frequencies; (**a**) TRU and TRM numbering structure (colored squares: measured points, yellow square: SLB antenna location); (**b**) test results for lower-edge elements; (**c**) test results for middle-edge elements; (**d**) test results for upper-edge elements.

**Figure 6:**
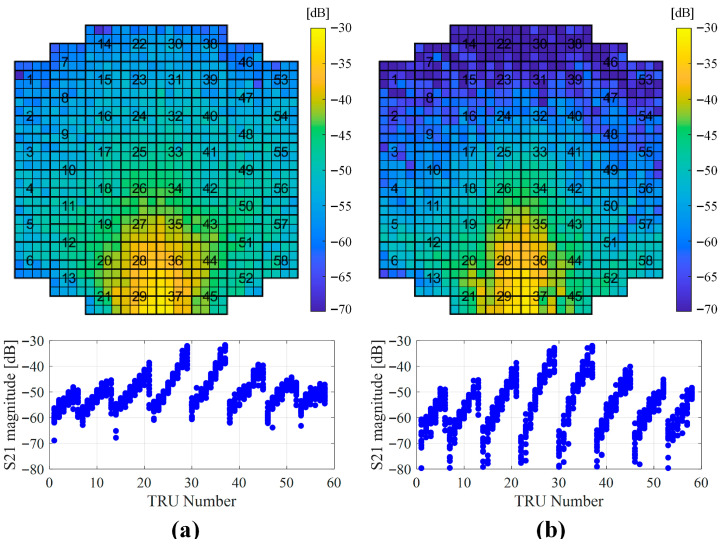
Coupling level test results for all TRM positions (blue dots in the below figures are measured coupling level of TRMs): (**a**) F81; (**b**) F121.

Figure 5 and Figure 6 shows the coupling level test result. Figure 5 shows the measurement result of some critical points for operational bandwidth, and Figure 6 shows the result of all channels for single frequency. Figure 5a shows numbering structure of TRM and TRU. To avoid confusion, authors divided the graphs into three sections: lower, middle, and upper edges. Selected points were critical points for determining detection threshold and RF input power to the pilot port. Frequencies in graphs were encoded for security reasons.

As can be seen in Figure 5b, TRU 37 is nearest point to the SLB and coupling level was higher than 45 dB. Figure 5c shows coupling level for TRU 54–57, and the level was maintained higher than 70 dB without some null frequencies. TRU 30–53 were far from the SLB antenna and had many nulls in the operation band, as depicted in Figure 5d. However, the coupling level was maintained above 75 dB. The estimated reason of the null points was the mutual coupling between radiating elements [27].

The BIT frequency was selected based on two key considerations. The first consideration was the coupling level. If a deep null occurs at a specific frequency or location, it becomes difficult to determine a reliable threshold. In this study, a threshold of −50 dBm was selected. Considering the radar processor’s maximum transmit power, receiver gain, and internal antenna path losses, a coupling level of at least −80 dB was required to ensure that the final received signal level exceeded −30 dBm. Frequencies F81 and F121 satisfied this condition, providing a stable coupling level above −80 dB.

The second consideration involved the radar’s operational bandwidth. While the antenna covers the frequency range from F1 to F201, specific situations necessitate dividing the frequencies into lower and upper bands, with F101 as the reference point.

Based on the critical point test result and above considerations, F81 and F121 were selected as BIT frequencies. Also, these frequencies were used for other BIT functions for APAR.

Using the same test configuration, the coupling levels of all TRMs at the selected BIT frequencies were measured, as shown in Figure 6. The coupling level gradually decreased with increasing distance from the SLB antenna. Some measurement points exhibited levels 4–5 dB lower than those obtained from the initial critical point tests; however, these values remain sufficient given the maximum output power of the SLB TRx (20 dBm) and the radar processor’s signal gain (35 dB). Moreover, as illustrated in Figure 5, stable coupling levels were maintained across most TRM locations, confirming the suitability of the selected BIT frequencies.

**Figure 7:**
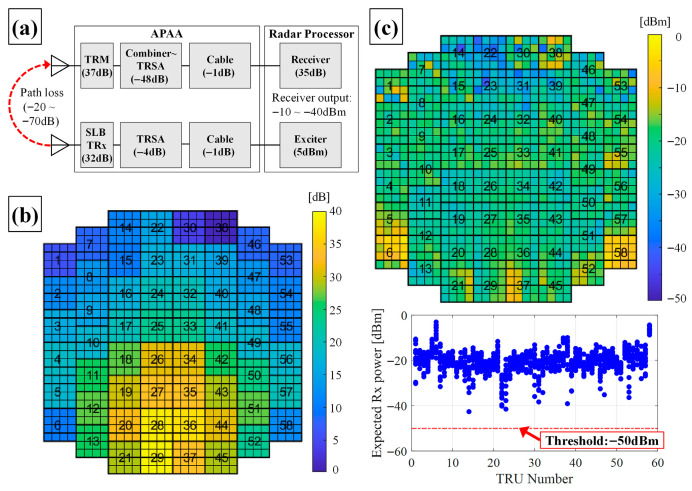
Simplified budget analysis and expected received signal level: (**a**) overall budget analysis result; (**b**) input attenuation level by TRU number; (**c**) expected received signal level at F121 (red dashed line: fault detection threshold).

Combining the RF budget analysis presented in Section 2.3 with the measurement results in Figure 6, the expected received signal level at the radar processor was derived, as summarized in Figure 7a. The corresponding input attenuation levels at the pilot port for each TRU are shown in Figure 7b, and the calculated received signal levels at F121 are presented in Figure 7c. Based on these results, the BIT detection threshold was confirmed to be –50 dBm.

Although the detection threshold could, in principle, be dynamically adjusted in response to environmental variations, a fixed threshold was adopted for implementation simplicity. Low software complexity is especially important in avionics systems, where reliability and determinism take priority. Moreover, because the APAA employs a water-cooling system, its thermal conditions remain stable, and the TRM output variation is limited to within 0.1 dB. Therefore, temperature-dependent threshold adjustments were deemed unnecessary.

### 2.5. Fault Detection Logic and Timing Control

**Figure 8:**
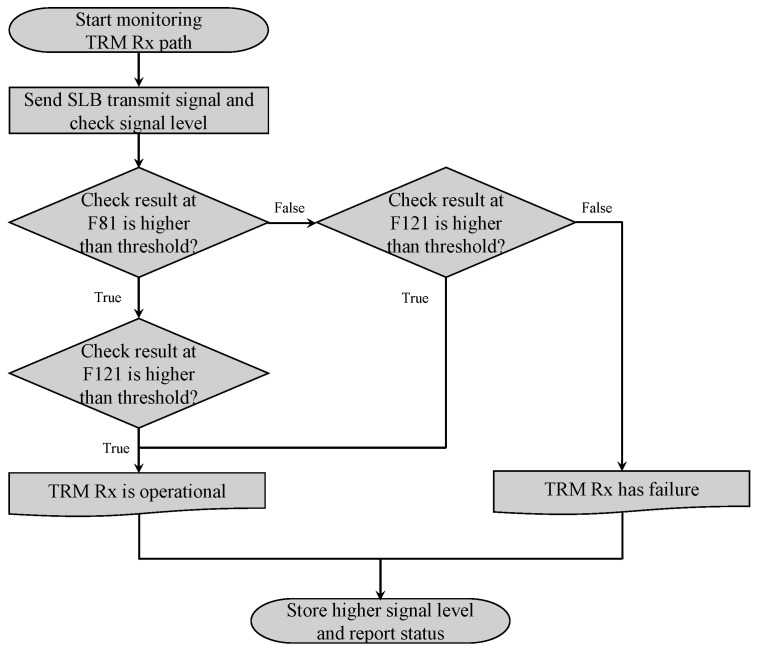
Fault detection logic for TRM diagnostics.

The fault detection logic is illustrated in Figure 8. During the BIT operation, the radar processor sequentially activated the receive paths of the TRMs while enabling the transmit path of the SLB TRx module. The radar processor then transmitted a test signal to the pilot port of the APAA.

Simultaneously, the APAR software monitored the received signal level within the radar processor. As previously described, the APAR employed two BIT frequencies and evaluated the signal levels of all TRMs at both frequencies. If the measured signal levels at both frequencies fell below the predefined detection threshold, the APAR identified the corresponding TRM as faulty. Upon completion of diagnostics, regardless of whether a fault was detected, the higher of the two measured signal levels at F81 and F121 was stored as the BIT result for that TRM.

From the perspective of test logic implementation, two aspects were considered. The first is impedance variation under different operational conditions. In controlled environments such as anechoic chamber or rooftop laboratory tests, external conditions remain stable, resulting in consistent signal levels. However, in airborne operational environments, temperature fluctuations and varying electromagnetic conditions can cause impedance changes in individual elements, occasionally leading to abrupt signal drops. To enhance reliability under such conditions, measurements were performed at two frequencies.

The second aspect concerns frequency band operation. As discussed in Section 2.4, the radar may, in certain situations, operate exclusively within either the lower or upper frequency band. In such cases, the measurement obtained at a single operating frequency was used as the final BIT result.

**Figure 9:**
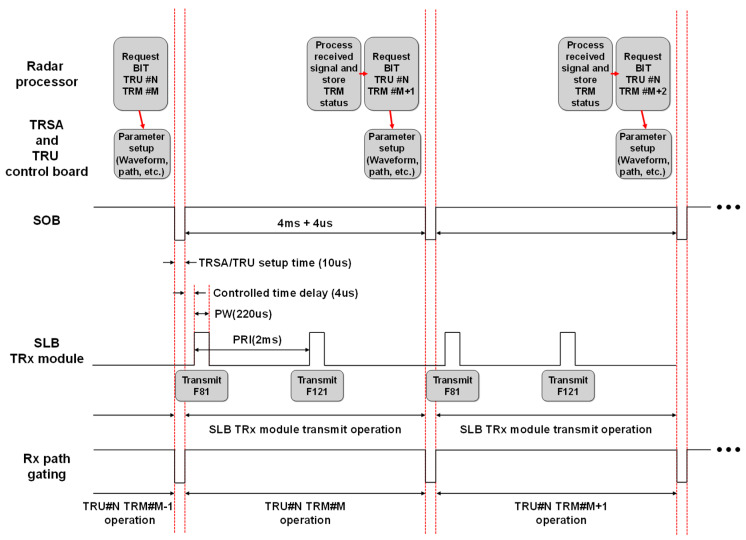
The control timing diagram of the APAA fault detection function.

For operating fault detection function for the APAR system level, the control timing was designed as shown in Figure 9. The radar processor first sent control packet to APAA in order to inspect the receive path of the TRM. The control packet contained the APAA control information such as the TRSA path control command, the TRM location to be tested, the number of pulses to be transmitted, pulse repetition interval (PRI), and pulse width (PW). The antenna controller parsed the received control packet and delivered it to TRSA and TRU control board while initiating preparation for command execution one start of burst (SOB) in advance.

The SOB signal served as the primary synchronization reference for all line-replaceable units (LRUs) within the APAR and was generated by the waveform generator in the radar processor. All preparatory operations were completed before the falling edge of the SOB signal.

During the 10 µs interval when the SOB signal remains low, the TRSA configured the test path and pulse parameters required for the SLB TRx module’s pulsed RF transmission. Concurrently, the TRU control board configured the designated TRM as instructed by the antenna controller. When the SOB signal rose, the radar processor transmitted a 5 dBm RF signal to the APAA’s pilot port, which was attenuated according to the levels specified in Figure 7. Each SOB cycle corresponded to one TRM test sequence, and two pulses at the two BIT frequencies (F81 and F121) were transmitted for evaluation. The PRI and PW were set to 2 ms and 220 µs, respectively.

A controlled timing delay of 4 µs was introduced between the rising edge of the SOB signal and the gating pulse of the SLB TRx module. This delay was adjustable via a control packet sent by the radar processor. The input signal was routed through the TRSA to the SLB TRx module, amplified, and radiated by the SLB antenna. The TRM under test received this signal, while all other TRMs remained inactive during BIT operation. The radar processor then measured the received signal level from the Rx Sum channel of the APAA and determined the TRM’s status according to the fault detection logic defined in Figure 8. Simultaneously, the radar system configured the parameters required for testing the next TRM in sequence.

## 3. Test Results

**Figure 10:**
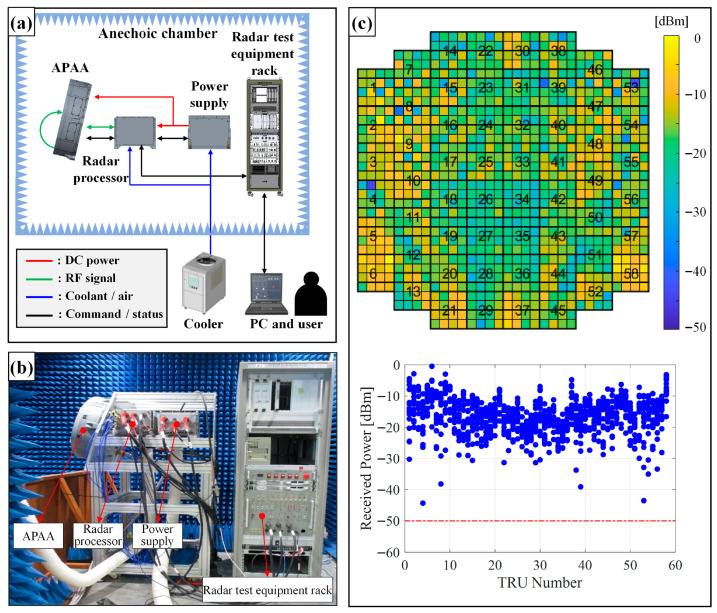
Initial APAR-level test environment and test result: (**a**) test setup; (**b**) actual test scenario; (**c**) test result (red dashed line: detection threshold, blue dots: received power level of TRMs).

Figure 10a,b illustrate the APAR-level test setup used to verify the TRM BIT function. To confirm and adjust the detection threshold, the initial tests were conducted inside the anechoic chamber.

In this setup, the radar test equipment rack controlled the radar processor, simulating the aircraft avionics environment by providing corresponding command data. A control PC, located outside the chamber, allowed the user to operate the APAR and analyze the results. The power supply was an actual radar LRU, mounted within the aircraft avionics bay, responsible for converting AC voltage to the appropriate DC levels for the APAA and radar processor. Cooling was provided by a combination of air and liquid supplied from an external cooler.

To minimize uncertainties caused by hardware performance degradation, newly manufactured APAA and radar processor units were used during testing.

As shown in Figure 10c, the measured signal levels closely matched the expected values from Section 2.5, confirming that the detection threshold was appropriately established.

**Figure 11:**
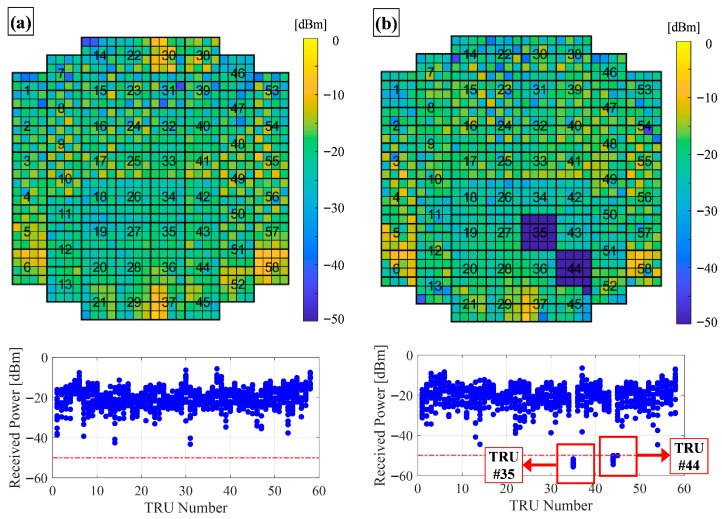
APAR-level field test results (red dashed line: detection threshold, blue dots: received power level of TRMs.): (**a**) aircraft test results; (**b**) rooflab test results with intentionally damaged TRUs.

Following the initial anechoic chamber tests, the radar software was updated for actual APAR flight models, which were then evaluated under various conditions, including a system integration laboratory, a flying testbed, and an operational fighter prototype [28]. Selected test results are shown in Figure 11. Figure 11a presents aircraft test results, while Figure 11b shows rooflab tests, where two TRUs were intentionally damaged. Across these tests, received signal levels were consistent among different APAA units, and signal levels fell below the detection threshold only when a TRU was non-operational. These results demonstrate that the implemented fault detection method functions reliably across multiple environments and with different LRUs.

Statistical analysis was conducted following field deployment based on collected data of false alarm occurrences and associated TRM locations for each flight sortie. Table 2 summarizes the results from 10 flight test sorties.

As indicated in Table 2, false alarms occurred for less than one minute per sortie, and fewer than two TRMs were erroneously flagged in each case. Overall, false alarms were observed in only 0.26% of total operation hours. Considering the complexity of monitoring hundreds of TRMs and the limited frequency of BIT checks during critical radar operation modes, these results indicate a high level of reliability for the implemented diagnostic method.

## 4. Discussion

This article presented the design and implementation of a TRM diagnostic function on existing airborne APAA hardware. To realize the TRM BIT, the authors utilized the SLB channel as a peripheral probe and conducted APAA-level tests to evaluate the coupling between the SLB antenna and the TRMs. Based on the coupling measurements, BIT frequencies were selected, and the fault detection threshold was determined using both the coupling results and the RF budget analysis. Input attenuation levels were carefully set to prevent damage to the TRM receive paths. On the software side, a simple two-frequency test logic was developed, and the associated control timing sequences were implemented on the existing hardware.

The initial APAR-level tests demonstrated good agreement with theoretical expectations, and subsequent field tests confirmed that the proposed method can accurately isolate faulty TRUs and TRMs.

Following APAR deployment, the false alarm rate was evaluated over ten flight sorties, revealing a reliably low false alarm incidence and confirming the robustness of the BIT function.

The proposed method is expected to be applicable to other existing APAAs equipped with SLB antennas and associated TRM measurement paths. Beyond airborne APARs, ground-based fire-control and shipborne multifunction radars also employ SLB channels, suggesting broad applicability of this approach. By leveraging existing hardware, modifications are minimized, and the implementation cost of the BIT function is reduced.

However, when extending this method to large-scale S-band or C-band radars, the increased physical distance between the SLB antenna and the TRMs may significantly affect coupling. Therefore, a careful RF budget analysis (Section 2.3) and coupling level measurements (Section 2.4) are essential prior to implementation. Appropriate detection thresholds can then be established through accurate analysis and prediction. Additionally, BIT reliability can be improved by adjusting the SLB antenna output to maintain received signal levels across TRMs within a narrow range.

While this method relies on signal level measurements and is thus limited to assessing the operational status of TRMs, further enhancements are possible. If the phase information of each TRM can be accurately acquired, an in situ calibration function could be implemented, maximizing APAR system availability. Achieving this would require upgrading the SLB TRx module to provide increased output power and controllable adjustment capability. Future work will focus on modifying the SLB TRx module and developing phase measurement procedures to enable in situ calibration functionality.

## Figures and Tables

**Table 1:** Signal injection chain budget analysis results (path loss between SLB antenna and TRM is median value).

APAA Submodule	Components	Gain (dB)	Cumulative Gain (dB)	Signal Input (dBm)	Signal Output (dBm)
TRSA (Pilot path)	SPDT	−1.5	−1.5	−20	−21.5
	SPDT	−1.5	−3	−21.5	−23
	Circulator	−1	−4	−23	−24
SLB TRx Module	Circulator	−1	−5	−24	−25
	Fixed attenuator	−1.5	−6.5	−25	−26.5
	LNA	19	12.5	−26.5	−7.5
	Fixed attenuator	−1.5	11	−7.5	−9
	LNA	19	30	−9	10
	Fixed attenuator	−1.5	28.5	10	8.5
	Circulator	−1	27.5	8.5	7.5
Path loss	Path loss	−40	−12.5	7.5	−32.5
TRM	Circulator	−1	−13.5	−32.5	−33.5
	Limiter	−1	−14.5	−33.5	−34.5
	LNA	36	21.5	−34.5	1.5
	MFC	3	24.5	1.5	4.5
Power combiner	Power combiner	−32	−7.5	4.5	−27.5
TRSA (Receive path)	Monopulse comparator	−8	−15.5	−27.5	−35.5
	Circulator	−1	−16.5	−35.5	−36.5
	VVA	−3	−19.5	−36.5	−39.5
	Fixed attenuator	−3	−22.5	−39.5	−42.5
	LPF	−1	−23.5	−42.5	−43.5

**Table 2:** False alarm statistics across 10 flight test sorties.

Sortie Number	Flight Time (min)	False Alarm Time (min)	TRB(TRM)
1	137	1	32(12), 40(13)
2	231	0.5	30(1), 32(4)
3	187	1	47(15)
4	193	0	
5	190	0	
6	204	1	39(14)
7	200	0	
8	204	0.5	23(8)
9	179	1	22(3)
10	134	0	
Total	1859	5	-

## Data Availability

The original contributions presented in this study are included in the article. Further inquiries can be directed to the corresponding author.
